# Hemoperitoneum in advanced abdominal pregnancy with a live baby: a case report

**DOI:** 10.1186/1756-0500-7-106

**Published:** 2014-02-25

**Authors:** Dismas Matovelo, Nhandi Ng’walida

**Affiliations:** 1 Department of Obstetrics and Gynecology, Catholic University of Health and Allied sciences, P.O. BOX 1464, Mwanza, Tanzania; 2 Department of Obstetrics and Gynecology, Bugando Medical Centre, P.O. BOX 1370, Mwanza, Tanzania

**Keywords:** Hemoperitoneum, Advanced abdominal pregnancy, Laparotomy, Live baby

## Abstract

**Background:**

Abdominal pregnancy is a rare condition which is usually missed during prenatal assessment particularly in settings lacking routine ultrasound surveillance. We report a case of abdominal pregnancy at 32 weeks, which is most likely to have been a tubal abortion with secondary implantation, leading to delivery of a healthy baby girl weighing 1.7 kg.

**Case presentation:**

A 22-year-old woman, gravid 3 para 2 was referred to our centre from a district hospital with complaint of generalized abdominal pain and reduced fetal movements. Although the initial abdomino-pelvic ultrasound done at our centre was read as normal, there was subsequently a strong clinical suspicion of abdominal pregnancy, which was confirmed by a second ultrasound. The patient underwent laparotomy and was found to have an intact uterus with a viable fetus floating in the abdominal cavity without its amniotic sac and with hemoperitoneum of 1litre. The baby was extracted successfully; the placenta was found to be deeply implanted on the right cornual side extending to the fundus superiorly. Wedge resection of the cornual area and fundus was performed to remove the placenta. Intraoperatively, one unit of blood was transfused due to severe anemia prior to surgery. Both the mother and the baby were discharged home in good condition.

**Conclusion:**

Abdominal pregnancy can be missed prenatally even when an imaging (ultrasound) facility is available. Emphasis should be placed on clinical assessment and thorough evaluation of patients.

## Background

Abdominal pregnancy is a rare form of ectopic pregnancy in which the implantation takes place in the peritoneal cavity outside the fallopian tube [1]. Despite the use of ultrasound, the diagnosis of abdominal pregnancy is usually missed during antenatal care. It occurs either as a result of tubal abortion or rupture, known as secondary abdominal pregnancy, or more rarely, as a direct implantation on the peritoneum, with normal fallopian tubes, normal ovaries, or without tubal fistula, known as primary abdominal pregnancy [2].

There is a high maternal morbidity and mortality associated with abdominal pregnancy. Its management depends on the gestational age and site where the placenta is attached [1,3]. We report on a successful operative delivery of a healthy baby in a woman with abdominal pregnancy at 32 weeks with hemoperitoneum in whom the diagnosis was missed during antenatal care and initial ultrasound.

## Case presentation

A 22-year-old woman, gravida 3 para 2, was admitted to our labour ward as a referral from Magu District hospital at 32 weeks with a one week history of generalized abdominal pain, reduced fetal movements and dysuria. She had been seen initially for antenatal care at a gestational age of 16 weeks, and had a total of two antenatal visits. Her pregnancy was thought to be uncomplicated during these visits. On admission to the labour ward, her vital signs were: blood pressure of 120/80 mmHg, pulse 110 beats/minutes and respiratory rate of 18 breaths/minute. Her abdomen was distended with a fundal height corresponding to 32 weeks with hypogastric tenderness. Fetal heart rate could not be established by fetoscope. Her laboratory results showed hemoglobin of 6 g/dl and urinalysis with 20 leukocytes per high power field. An ultrasound examination revealed a live intrauterine pregnancy at 31 weeks of gestation but the lie of the fetus and location of the placenta were not established. The impression of the admitting doctor was of an intrauterine pregnancy complicated by anemia and urinary tract infection (UTI). The patient received iron supplements, oral nitrofurantoin 500 mg every 8 hours for 5 days and paracetamol; her pain subsided, and she was admitted to the ward for follow-up and further investigation. Two days later, the case was reviewed by senior doctors on the ward, at that time, the patient continued to have generalized abdominal pain, and on examination she was found to have generalized abdominal tenderness with rebound tenderness and easily palpable fetal parts. A provisional diagnosis of abdominal pregnancy was made and an urgent repeat abdomino-pelvic ultrasound examination was performed. The ultrasound revealed an empty bulky uterus, a viable fetus in the abdominal cavity at 31 weeks, and a placenta attached anterior to the uterus. The decision was made to do an emergency laparotomy based on clinical features of peritonitis. Under general anesthesia and through sub-umbilical midline incision, the uterus was found to be empty and small. A fetus was found floating in the abdominal cavity without an amniotic sac. There was hemoperitoneum of about 1 litre. A baby girl weighing 1.7 kg was extracted with Apgar scores of 8 and 10 at the first and fifth minutes respectively. The placenta was adherent to the omentum and to the right cornual area extending to the fundus superiorly. Both ovaries and the right fallopian tube were healthy. The left fallopian tube had adhesive bands and fimbrial phimosis. Suctioning of the hemoperitoneum was performed, omental adhesions were released, and cornual resection was done at the site where the placenta had implanted. Intraoperatively one unit of blood was given as the patient was severely anemic prior to surgery, and a tourniquet was applied to minimize uterine vessel bleeding prior to resection. The placenta was completely removed and hemostasis was achieved after the removal of the tourniquet 20 minutes later. By day 3 post-laparotomy, the mother could mobilize fully, eat, drink and had normal bowel movements and micturition habits. The baby was breast feeding and eliminating well.

The patient was discharged on day 7 after suture removal with iron and folate tablets. During her stay in the hospital, she was counseled on the possibility of recurrence of abdominal pregnancy. She was told to come back for follow-up after 2 weeks, but the patient was lost to follow-up.

### Discussion

Although ultrasound can be useful in diagnosing abdominal pregnancy, it is easier to appreciate the abdominal pregnancy during the end of the first trimester or early in the second trimester, when the pelvic organs are best visualized. In advanced abdominal pregnancy visualization of pelvic organs becomes limited. Other diagnostic tools include plain abdominal radiography and magnetic resonance imaging (MRI) [1,4]. In our patient, initially a diagnosis of advanced abdominal pregnancy was missed by both clinical findings and imaging studies. Thus the diagnosis requires clinical suspicion, as ultrasound will miss almost 50% of abdominal pregnancies in the absence of clinical suspicion [4,5].

There are several risk factors for abdominal pregnancy which include previous history of tubal pregnancies, pelvic inflammatory disease, tubal sterilization, tubal infertility, tubal reconstructive surgery and conceiving with intrauterine contraceptive device (IUCD) in situ [2]. In our case, the possible risk factor could be pelvic inflammatory disease, suspected due to the presence of adhesion bands and fimbrial phimosis on her left fallopian tube.

Early diagnosis of abdominal pregnancy depends on a high index of suspicion from the antenatal care providers [3,5]. Several symptoms have been established to suggest the presence of abdominal pregnancy. These symptoms include recurrent abdominal pain, painful fetal movements, and easily palpable fetal parts. Difficulty in establishing fetal lie and presenting part is another sign which should raise suspicion for abdominal pregnancy. Signs and symptoms of peritoneal irritation can be an additional indicator, as this can suggest hemoperitoneum as in our patient [1,3,6,7].

It is vital for the diagnosis of abdominal pregnancy to be made early in pregnancy. Morbidity and mortality are largely due to massive hemorrhage that may arise from complete or partial placental separation [1,8]. The placenta can implant at different sites including but not limited to the uterine wall, adnexal, bowel, omentum, liver, spleen and pouch of Douglas, and the placenta may separate anytime during pregnancy leading to hemorrhage and anemia as illustrated our case. In our case, we found a live fetus floating in the peritoneal cavity due to a ruptured amniotic sac. The survival of this fetus could be in part due to a timely decision for laparotomy, as well as to a placenta that was partially adherent to the fundus and left cornual end which provided enough blood supply and oxygenation to the fetus. In most cases of advanced abdominal pregnancy, fetal outcome tends to be good if the fetus is covered by the amniotic sac [8].

Our patient required emergency laparotomy due to the suspicion of hemoperitoneum, as unstable maternal hemodynamic status is among the criteria for surgery. Other criteria for surgery include fetal congenital malformations, fetal viability, gestational age at presentation and availability of facilities for neonatal care. If the fetus is alive, surgery may be performed regardless of gestational age or fetal status due to difficulties in predicting placental separation leading to massive hemorrhage. If the fetus is dead, surgery is necessary due to the risk of disseminated intravascular coagulopathy (DIC) or sepsis, although a period of 4–8 weeks may be allowed for observation to allow atrophy of placental vessels [3,9].

In our patient, the placenta was easily removed by performing wedge resection at the right cornual end and uterine fundus. Otherwise, the placenta should be left in situ to minimize the risk of massive hemorrhage. Attempts to remove the placenta should only be done when the surgeon is able to ligate all the placental vessels [7,10].

## Conclusions

The important message of this case report is that abdominal pregnancy can be missed during antenatal care and by ultrasound. Emphasis should be placed on clinical assessment and thorough evaluation of patients. Also, vigilant management of a patient with abdominal pregnancy is required to achieve a favorable maternal and fetal outcome.

## Consent

A written informed consent was obtained from the patient for publication of this case report. A copy of the written consent is available for review by the Editor-in-Chief of this journal.

## Competing interests

The authors declare that they have no competing interests.

## Authors' contributions

DM performed the surgery, wrote and reviewed the manuscript. NN reviewed the patient prior to surgery and revised several drafts of the manuscript. Both authors read and approved the final manuscript.
